# Recent advances in systemic therapy for hepatocellular carcinoma

**DOI:** 10.1186/s40364-021-00350-4

**Published:** 2022-01-09

**Authors:** Huajun Zhang, Wuyang Zhang, Longying Jiang, Yongheng Chen

**Affiliations:** 1 Department of Oncology, NHC Key Laboratory of Cancer Proteomics, Laboratory of Structural Biology, Xiangya Hospital, Central South University, Changsha, 410008 Hunan China; 2 Clinical skills training center, Xiangya Hospital, Central South University, Changsha, 410008 Hunan China; 3 Department of Pathology, Xiangya Hospital, Central South University, Changsha, 410008 Hunan China; 4 National Clinical Research Center for Geriatric Disorders, Xiangya Hospital, Central South University, Changsha, 410008 Hunan China

**Keywords:** Hepatocellular carcinoma, Molecular targeted therapy, Immunotherapies, Combination

## Abstract

Hepatocellular carcinoma (HCC) is one of the most common and lethal malignant tumors in the world. Therapeutic options for advanced HCC are limited. Systemic treatment, especially with conventional cytotoxic drugs, is usually ineffective. For more than a decade, sorafenib has been the only systemic drug that has been proven to be clinically effective for treating advanced HCC. However, over the past three years, the rapid progress of molecular targeted therapies has dramatically changed the treatment landscape for advanced HCC. Immune checkpoint therapies are now being incorporated into HCC therapies, and their combination with molecular targeted therapy is emerging as a tool to enhance the immune response. In this review, we summarize the development and progress of molecular targeted agents and immunotherapies in HCC.

## Background

**Fig. 1:**
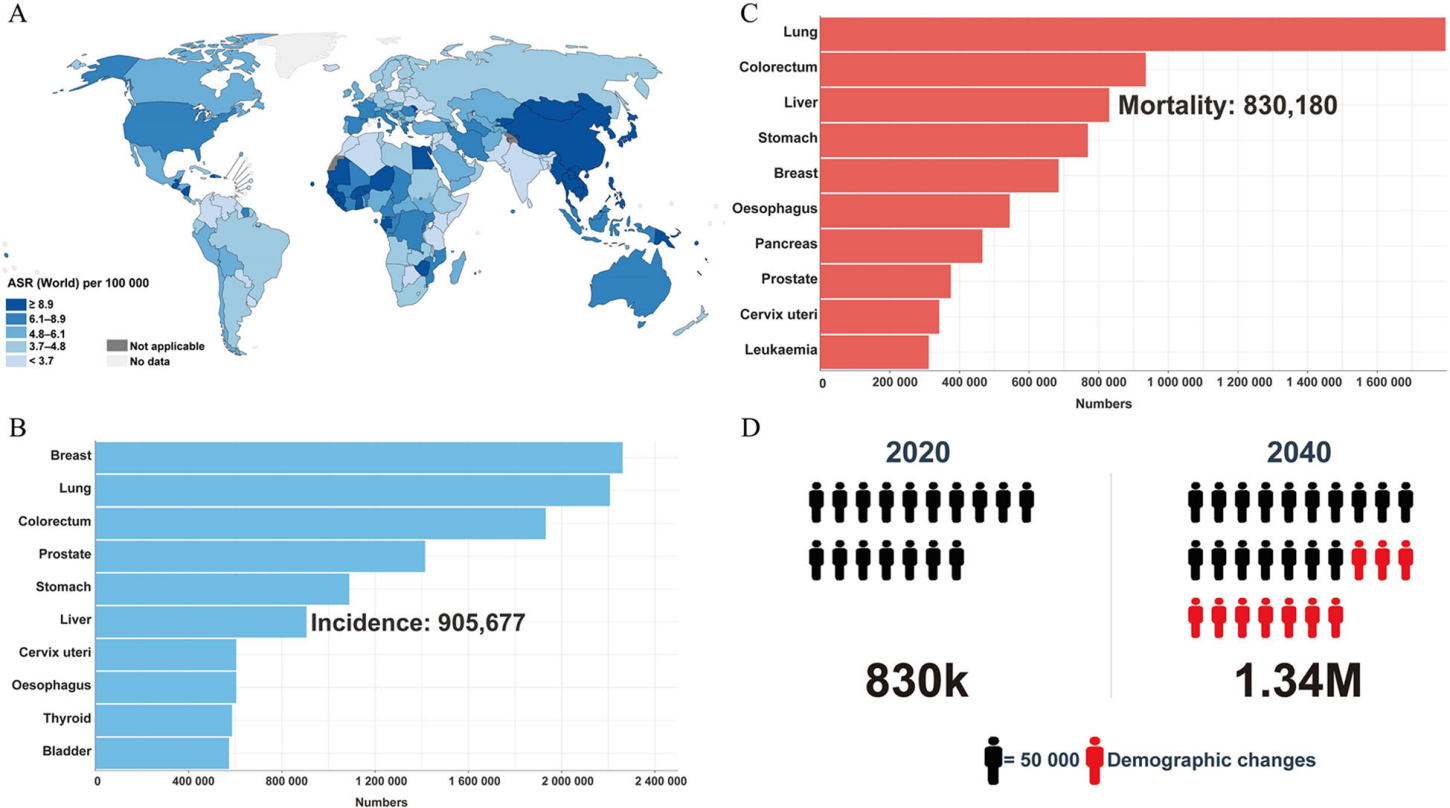
Worldwide Epidemiology of Liver Cancer in 2020. Data source: GLOBOCAN 2020 (http://gco.iarc.fr/). (**A**) The estimated age-standardized incidences of liver cancer worldwide in 2020. (**B**) Bar charts of the estimated number of incident cases worldwide. (**C**) Bar charts of the estimated number of deaths worldwide. (**D**) WHO estimated the number of deaths from liver cancer from 2020 to 2040

Worldwide, liver cancers are predicted to rank sixth for incident cases and be the third leading cause of cancer-related death in 2020, with approximately 905,677 new cases and 830,180 deaths each year (Fig. 1A-C) [1]. Based on annual estimates, the World Health Organization predicts that more than 1.3 million people will die from liver cancer by 2040 (Fig. 1D) [2]. HCC is the most frequently occurring tumor among all primary liver cancers, accounting for 75%-85% of cases [3]. HCC is usually diagnosed at an advanced stage, for which there remain limited effective treatment options [4]. When applying palliative treatment at an advanced stage, the median post-diagnostic survival ranged from 6 to 12 months [5]. Systemic treatments may benefit patients with advanced-stage HCC [6].

**Fig. 2:**
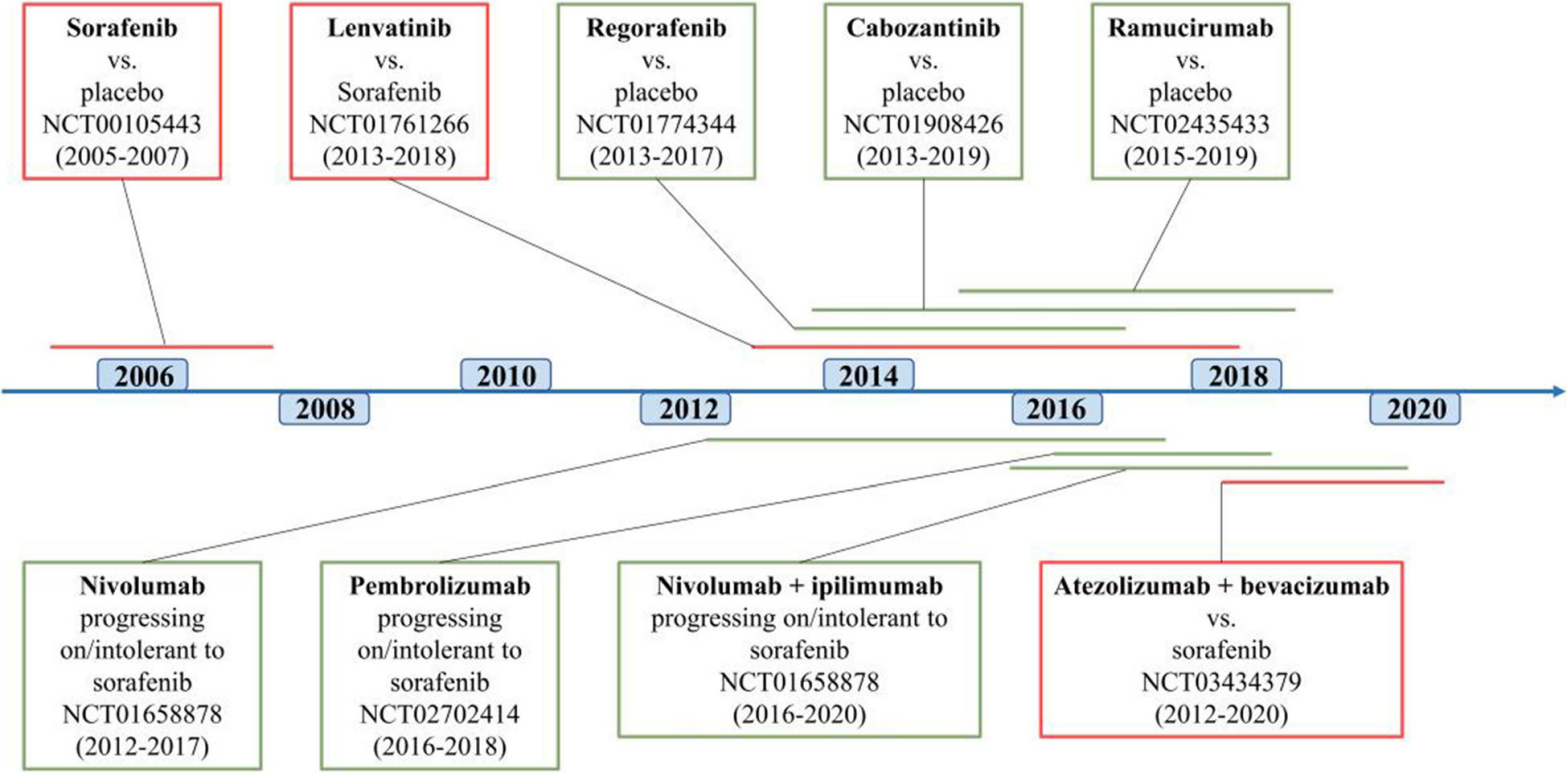
Currently approved drugs for advanced HCC and timeline of pivotal clinical trials. The lines along the timeline indicate the time from the actual study start to FDA approval. The red boxes represent first-line therapies, and the green boxes represent second-line therapies

Until 2007, there were no effective treatment options for patients diagnosed with advanced-stage disease or patients who transitioned into advanced-stage disease after other treatments failed [7]. Sorafenib is the first and only systemic drug approved by the U.S. Food and Drug Administration (FDA) as standard treatment for advanced HCC between 2007 and 2016. However, rapid advances during the last 3 years have led to the approval of other molecular targeted drugs and several immune checkpoint inhibitors (ICIs), which have been added as therapeutic tools for advanced HCC (Tables 1, 2 and 3). In addition, the recent successful combination of atezolizumab and bevacizumab marks an important change in the first-line treatment of HCC (GO30140, NCT NCT02715531) [8]. To date, systemic therapy for advanced HCC includes molecular targeted therapy, immune checkpoint inhibitors, or a combination of both (Fig. 2).

**Table 1:** Clinical trials of molecular targeted therapy for advanced HCC

Drug	Setting	Name/ID	Phase	Target	Results	Approval Status
Sorafenib	1st	SHARP	III	Multikinases	mOS 10.7 vs. 7.9 months (HR 0.69, *p*<0.001)	First-line, 2008
		NCT00492752	III		mOS 6.5 vs. 4.2 months (HR 0.68, *p*=0.014)	/
Lenvatinib	1st	REFLECT	III	Multikinases	mOS 13.6 vs. 12.3 months (compared to sorafenib, HR 0.92)	First-line, 2018
Erlotinib	1st	SEARCH	III	EGFR	mOS 9.5 vs. 8.5 months (compared to sorafenib)	/
Brivanib	1st	BRISK-FL	III	VEGFR, PDGFR, FGFR	mOS 9.5 vs. 9.9 months (compared to sorafenib)	/
Sunitinib	1st	NCT00699374	III	VEGFR, PDGFR	mOS 7.9 vs. 10.2 months (compared to sorafenib)	/
Linifanib	1st	NCT01009593	III	VEGFR, PDGFR	mOS 9.1 vs. 9.8 months	/
Everolimus	2nd	EVOLVE-1	III	mTOR	mOS 7.6 vs. 7.3 months	/
Tivantinib	2nd	NCT01755767	III	c-MET	mOS 8.4 vs. 9.1 months	/
Regorafenib	2nd	RESORCE	III	Multikinases	mOS 10.6 vs. 7.8 months (HR 0.63, *p*<0.001)	Second-line, 2017
Cabozantinib	2nd	CELESTIAL	III	Multikinases	mOS 10.2 vs. 8 months (HR 0.76, *p*=0.005)	Second-line, 2019
Ramucirumab	2nd	REACH-2	III	VEGFR2	mOS 8.5 vs. 7.3 months (HR 0.71, *p*=0.0199)	Second-line, 2019
Tepotinib	1st	NCT01988493	Ib/II	c-MET	mTTP 2.9 vs. 1.4 months (compared to sorafenib, HR 0.42, p=0.0043)	/
	2nd	NCT02115373	Ib/II		RP2D 500mg, 12-week PFS 63.3% (*p* < 0.0001)	/
Capmatinib	1st	NCT01737827	II	c-MET	/	/
Fisogatinib (BLU554)	1st/2nd	NCT02508467	I	FGFR4	/	/
Roblitinib (FGF401)	/	NCT02325739	I/II	FGFR4	/	/
H3B-6527	2nd	NCT02834780	I	FGFR4	/	/
Tivozanib	1st	NCT01835223	I/II	Multikinases	ORR 21%, mPFS 6 months, mOS 9 months, did not proceed to stage 2	/
Donafenib	1st	ZGDH3	III	Multikinases	mOS 12.1 vs. 10.3 mo (compared to sorafenib, HR 0.831, *p*=0.0363)	/
Apatinib	2nd	AHELP	III	Multikinases	mOS 8.7 vs. 6.8 mo (HR 0.785, *p*=0.0476)	/

Abbreviations: *ORR* objective response rate, *mOS* median overall survival, *mPFS* median progression-free survival, *mTTP* median time to progression, *RP2D* recommended phase 2 dose

**Table 2:** ICI monotherapy for advanced HCC

ICI	Setting	Target	Phase	Name/NCT No.	Results	Approval Status
Tremelimumab	1st/2nd	CTLA-4	II	NCT01008358	/	/
Durvalumab	1st/2nd	PD-L1	I/II	NCT01693562	/	/
	2nd		III	NCT03847428	/	/
Avelumab	2nd	PD-L1	II	NCT03389126	ORR 10%, DCR 73.3%, mTTP 4.4 months, mOS 14.2 months	/
Nivolumab	2nd	PD-1	I/II	CheckMate-040	ORR 20%, mPFS 4.0 months	Conditional second-line, 2017
	1st		III	CheckMate-459	ORR 15%, mPFS 16.4 months	/
Pembrolizumab	1st/2nd	PD-1	II	KEYNOTE-224	ORR 17%, mOS 12.9 months, mPFS 4.9 months, mTTP 4.9 months	Conditional second-line, 2018
					1st setting: ORR 16%, DCR 57%, mOS 17 months	/
	2nd		III	KEYNOTE-240	ORR 18.3%, DCR 61.9%, mOS 13.9 months, mPFS 3.3 months	/
	2nd		III	KEYNOTE-394	/	/
	2nd		III	KEYNOTE-937	/	/
Tislelizumab	1st	PD-1	III	RATIONALE-301	/	/
Camrelizumab	2nd	PD-1	II	NCT02989922	ORR 14.7%, 6-month OS 74.4%	/

Abbreviations: *DCR* disease control rate, *ORR* objective response rate, *mOS* median overall survival, *mPFS* median progression-free survival, *mTTP* median time to progression

**Table 3:** ICI combination therapy for advanced HCC

Regimen	Setting	Phase	Name/NCT No.	Results	Approval Status
ICI + MKI					
Nivolumab + Ipilimumab	2nd	I/II	CheckMate-040	ORR 32%, DCR 54%, mOS 22.2 months	Conditional second-line, 2020
	1st	III	CheckMate-9DW	/	/
Nivolumab + Sorafenib	1st	II	NCT03439891	/	/
Nivolumab + Lenvatinib	1st	Ib	NCT03418922	/	/
Nivolumab + BMS986253	1st	II	NCT04050462	/	/
Nivolumab + Mogamulizumab	2nd	I/II	NCT02705105	/	/
Nivolumab + Galunisertib	2nd	Ib/II	NCT02423343	/	/
Nivolumab + Relatlimab	2nd	II	NCT04567615	/	/
Nivolumab + Cabozantinib	neoadjuvant	Ib	CaboNivo/NCT03299946	/	/
Pembrolizumab + Regorafenib	1st	Ib	NCT03347292	/	/
Pembrolizumab + Lenvatinib	1st	Ib	KEYNOTE524/NCT03006926	ORR 46%, mPFS 9.3 months, mOS 22 months	/
	1st	III	LEAP-002/NCT03713593	/	/
Atezolizumab + Bevacizumab	1st	Ib	GO30140	ORR 36%, mPFS 5.6 vs. 3.4 months (compared to atezolizumab monotherapy, *p*=0.011)	/
	1st	III	IMbrave150	ORR 29.8%, mOS 19.2 vs. 13.4 months (compared to sorafenib, HR 0.66, *p*=0.0009)	First-line treatment, 2020
Atezolizumab + Cabozantinib	1st	III	COSMIC-312/NCT03755791	/	/
Avelumab + Axitinib	1st	I	NCT03289533	ORR 31.8%, mPFS 3.8 months	/
Avelumab + Regorafenib	2nd	I/II	REGOMUNE/NCT03475953	/	/
Durvalumab + Cabozantinib	2nd	Ib	NCT03539822 (CAMILLA)	/	/
Durvalumab + Ramucirumab	2nd	I	NCT02572687	/	/
Durvalumab + tivozanib	1st	Ib/ II	DEDUCTIVE/NCT03970616	Ib: 2 of 7 achieving PR	/
Camrelizumab + Apatinib	1st	I	NCT02942329	ORR 50%, mPFS 5.8 months	/
	2nd	II	RESCUE/ NCT03463876	/	/
	1st	III	NCT03764293	/	/
Emibetuzumab + amucirumab	2nd	I/II	NCT02082210	/	/
ICI + ICI					
IBI310 + Sintilimab	1st	III	NCT04720716	/	/
Durvalumab + Tremelimumab	1st/2nd	I/II	NCT02519348	ORR 24%, mOS 18.7 months	/
	1st	III	HIMALAYA/NCT03298451	/	/

Abbreviations: *ICI* immune checkpoint inhibitor, *MKI* multikinase inhibitor, *ORR* objective response rate, *mOS* median overall survival, *mPFS* median progression-free survival, *mTTP* median time to progression, *PR* partial response

In this review, we present a summary of systemic drugs already approved for use in advanced HCC and those that have shown positive results in preclinical or clinical trials, providing a perspective on the future of advanced HCC therapies.

## Agents approved by the FDA

### Molecular targeted therapies

Over the last few decades, advances in molecular cell biology have greatly contributed to our knowledge of the molecular mechanisms of tumorigenesis and its progression, which in turn provides opportunities for the development of novel molecular targeted agents that inhibit molecular abnormalities as promising cancer treatments [9]. At present, molecular targeted therapy mainly includes tyrosine kinase inhibitors (TKIs) and/or monoclonal antibodies.

#### Sorafenib

Sorafenib is an oral TKI that targets the angiogenesis and proliferation pathways of tumors by blocking vascular endothelial growth factor receptor (VEGFR) 1–3, platelet-derived growth factor receptor (PDGFR) β-pathways, and Raf-MEK-ERK signaling, all of which participate in the pathogenesis of HCC (Fig. 4) [11, 12].

At ASCO 2007, the results of the SHARP (Sorafenib Hepatocellular Carcinoma Assessment Randomized Protocol; NCT00105443) were reported, which demonstrated the effectiveness of sorafenib against HCC. The primary endpoints were overall survival (OS) and time to symptomatic progression. Compared to the placebo group, the sorafenib treatment group had significantly prolonged OS (median OS 10.7 vs. 7.9 months, hazard ratio [HR]=0.69; 95% confidence interval [CI], 0.55–0.87; *p*<0.0001), while the time to symptomatic progression was not significantly different between the two groups (median time 4.1 vs. 4.9 months, HR=1.08, *p*=0.77). Statistically significant differences were found for secondary endpoints: the median time to radiologic progression improved by 3 months (5.5 vs. 2.8 months, *P*<0.001), and the sorafenib group had a higher disease-control rate than the placebo group (43% vs. 32%, *P* = 0.002). Common adverse effects observed in the sorafenib group included diarrhea, weight loss, hand-foot skin reactions, alopecia, anorexia, and voice changes, which were more frequent than those in the placebo group (*P*<0.001). Notably, 97% of the patients included in this trial were assessed as Child-Pugh liver function class A [13]. Based on these data, sorafenib was approved by the U.S. FDA in November 2007 for advanced HCC as first-line standard treatment.

Although sorafenib has a notable survival benefit in patients with advanced HCC, it has yet to be maintained. Owing to adverse events (AEs), many patients receiving sorafenib suffer from disease progression after dosage reductions or discontinuing treatment [14, 15]. In addition, 27% of patients in the SHARP trial had no initial response to sorafenib [13]. This reveals that resistance to sorafenib (both primary and acquired) greatly limits its survival benefit. In a study predicting survival in advanced HCC patients who permanently discontinued sorafenib, the median post-sorafenib survival was only 4.1 (95% CI 3.3-4.9) months. Liver decompensation, performance status (PS), tumor progression, and/or extrahepatic tumor spread were significantly associated with post-sorafenib survival [16].

#### Lenvatinib

As first-line treatment, most drugs subsequently tested in phase III trials failed to improve the prognosis compared to sorafenib; they also did not improve survival compared with placebo as second-line therapy. These drugs include erlotinib [17], brivanib [18], sunitinib [19], linifanib [20], everolimus [21], and tivantinib [22] (Fig. 4, Table 1). Insufficient antitumor activity, toxicity in a cirrhosis state, and inadequate patient selection were cited as reasons for these failures. Sorafenib has remained the sole effective first-line treatment option for over a decade until lenvatinib was finally proven to be non-inferior to sorafenib in terms of OS [23].

Lenvatinib is a small molecule inhibitor of VEGFR 1–3, fibroblast growth factor receptor (FGFR) 1–4, PDGFRα, KIT, and RET (Fig. 4) [24, 25]. Targeting the FGF signaling pathway in HCC differentiates lenvatinib from sorafenib.

In a multicenter, randomized, phase III, noninferiority trial (REFLECT; NCT01761266), lenvatinib exhibited noninferiority in overall survival compared with sorafenib (mOS, 13.6 vs. 12.3 months, HR=0.92, 95% CI, 0.79–1.06). All secondary efficacy endpoints in REFLECT were statistically significantly improved: the median progression-free survival (mPFS) was longer in the lenvatinib group than in the sorafenib group (mPFS, 7.3 vs. 3.6 months, HR=0.65, *p*< 0.0001); the median time to progression (mTTP) was 7.4 months in the lenvatinib group compared to 3.7 months in the sorafenib group (HR=0.61, *p*<0.0001); and the objective response rate was better (ORR, 18.8% vs. 6.5%, *p*<0.0001) based on the Response Evaluation Criteria in Solid Tumors (RECIST) version 1.1 after masked independent imaging review [23]. Based on the REFLECT results, the U.S. FDA approved lenvatinib for the first-line treatment of patients with advanced HCC on August 16, 2018 [26].

#### Regorafenib

Regorafenib is an oral inhibitor that inhibits many angiogenic and tumorigenic kinases, such as VEGFR1–3, tyrosine kinase with immunoglobulin and epidermal growth factor homology domain 2 (TIE2), PDGFRβ, FGFR1, B-RAF, RET, and KIT (Fig. 4) [27]. Regorafenib may exert a more far-reaching antiangiogenic activity owing to a combined blockade of the VEGFR2 and TIE2 pathways, which is supported by the results from a preclinical study by Tsai and Lee [28] showing a cooperative effect when combining anti-TIE2 and sorafenib, leading to an observed increase in overall survival in a melanoma model.

On April 27, 2017, the U.S. FDA expanded the indication for regorafenib as a second-line treatment for advanced HCC patients previously treated with sorafenib [29]. Approval was based on a randomized, placebo-controlled international phase III trial designed to assess the safety and efficacy of regorafenib in patients with HCC progressing during sorafenib treatment (RESORCE; NCT01774344) [30]. Regorafenib had a longer OS than placebo (mOS, 10.6 vs. 7.8 months, HR=0.63, *p*<0.0001) and prolonged progression-free survival and time to progression by mRECIST (mPFS 3.1 vs. 1.5 months, HR=0.46, *p*<0.0001; mTTP was 3.2 vs. 1.5 months, HR=0.44, *p*<0.0001)). The regorafenib group had an ORR of 11% versus 4% compared to the placebo group (*p*=0.0047), and the disease control rate (DCR) was 65% versus 35% (*p*<0.0001). Of note, the RESORCE trial was performed in patients who had progressed on prior sorafenib treatment; thus, the efficacy of regorafenib among sorafenib-intolerant patients has not yet been determined.

Additional analyses from the RESORCE trial suggested that regorafenib might offer clinical benefits independent of the latest sorafenib dose or the time to progression after previous sorafenib treatment and that the occurrence of AEs was independent of the last sorafenib dose [31]. Multiple clinical trials are currently evaluating the efficacy of regorafenib together with ICIs (Table 3).

#### Cabozantinib

Cabozantinib is a small-molecule tyrosine kinase inhibitor of MET and VEGFR 1-3, RET, KIT, AXL, and FLT3, all of which are associated with tumor pathogenesis (Fig. 4) [32, 33]. Cabozantinib has remarkable antitumor activity in HCC through dual inhibition of MET and VEGFR2 [33].

In an international, phase III double-blinded trial, 773 patients who progressed on no more than two prior systemic treatments including sorafenib were randomized to cabozantinib or placebo (CELESTIAL; NCT01908426). The primary endpoint of the CELESTIAL trial was that OS was significantly improved (mOS 10.2 vs. 8.0 months, HR=0.76, *p*=0.005). The secondary efficacy endpoints of the trial showed that cabozantinib significantly improved the PFS and ORR according to RECIST v1.1 (mPFS was 5.2 vs. 1.9 months, HR=0.44, *p*<0.001; ORR was 4% vs. <1%, *p*=0.009). Sixty-eight percent of patients in the cabozantinib group and 36% of patients in the placebo group experienced grade 3 or 4 AEs [34]. Based on these results, on January 14, 2019, the U.S. FDA approved cabozantinib for patients with HCC who had previously received sorafenib treatment [35]. Several trials evaluating the combinations of cabozantinib and ICIs in patients with advanced HCC are ongoing, including in combination with nivolumab and atezolizumab (Table 3).

Of note, in the CELESTIAL trial, 192 (27%) patients had received two previous systemic anticancer drugs, meaning that cabozantinib was used as third-line therapy. Within this subgroup, the OS was not significantly different (mOS 8.6 months vs. 8.6 months). However, cabozantinib improved the PFS (mPFS 3.7 vs. 1.9 months, HR=0.58, 95% CI, 0.41-0.83), suggesting that advanced HCC patients could use cabozantinib as a possible third-line option [34].

#### Ramucirumab

Ramucirumab, a humanized recombinant IgG1 monoclonal antibody, selectively binds to VEGFR-2, preventing activation of the VEGF pathway (Fig. 4) [36], and it showed anti-tumor activity in advanced HCC in a phase II trial [37]. A prospective study focusing on ramucirumab applied to treat the progression of advanced HCC while on sorafenib (REACH; NCT01140347) showed that ramucirumab did not lead to a significant improvement in OS compared to placebo (mOS 9.2 vs. 7.6 months, HR =0.87, *p*=0.14). However, in a subgroup with high baseline α-fetoprotein (AFP) concentrations (≥ 400 ng/mL), ramucirumab significantly prolonged the OS with a median OS of 7.8 months vs. 4.2 months (HR=0.674, *p*=0.006) [38].

To further investigate this result, the biomarker-driven REACH-2 trial (NCT02435433) was designed, evaluating ramucirumab versus placebo in advanced HCC patients with elevated AFP levels (≥400 ng/mL) who received prior sorafenib treatment. The primary endpoint of the REACH-2 trial showed that compared to the placebo group, OS was longer in the ramucirumab group (mOS 8.5 vs. 7.3 months, HR=0.71, *p*=0.0199). Ramucirumab also increased the PFS (mPFS 2.8 vs. 1.6 months, HR=0.452, *p*<0.0001), time to radiographic progression (mTTP 3.0 vs. 1.6 months, HR=0.427, *p*<0.0001), and disease control rate (59.9% vs. 38.9%, *p*=0.0006). REACH-2 was the first phase III trial to successfully screen for therapeutic candidates with biomarkers among patients with advanced HCC [39].

Based on these results, on May 10, 2019, the U.S. FDA authorized ramucirumab as a second-line treatment for HCC patients whose AFP was no less than 400 ng/mL [40].

### Monotherapy with immune checkpoint inhibitors

The success of anti-cytotoxic T lymphocyte associated antigen 4 (CTLA-4) antibodies in blocking immune checkpoints in advanced melanoma patients offers hope for immunotherapy of tumors. HCC carcinogenesis is typically accompanied by chronic liver inflammation, and HCC could be immunogenic [41]. In general, immune tolerance occurs in HCC mainly due to myeloid-derived suppressor cells (MDSCs), alterations of immune checkpoint molecules (such as CTLA-4 and programmed cell death protein-1 [PD-1]), and enrichment of T-regulatory cells (Tregs) [42]. The first evidence showing that ICIs could make a significant difference in HCC treatment came from a phase II trial in which the safety and antitumor activity of tremelimumab (CTLA-4 blockade) supported further studies in patients with advanced HCC who developed HCV-induced cirrhosis [43] (Table 2). In addition, other clinical trials of ICI monotherapy for HCC have been performed (Table 2).

#### Nivolumab

**Fig. 3:**
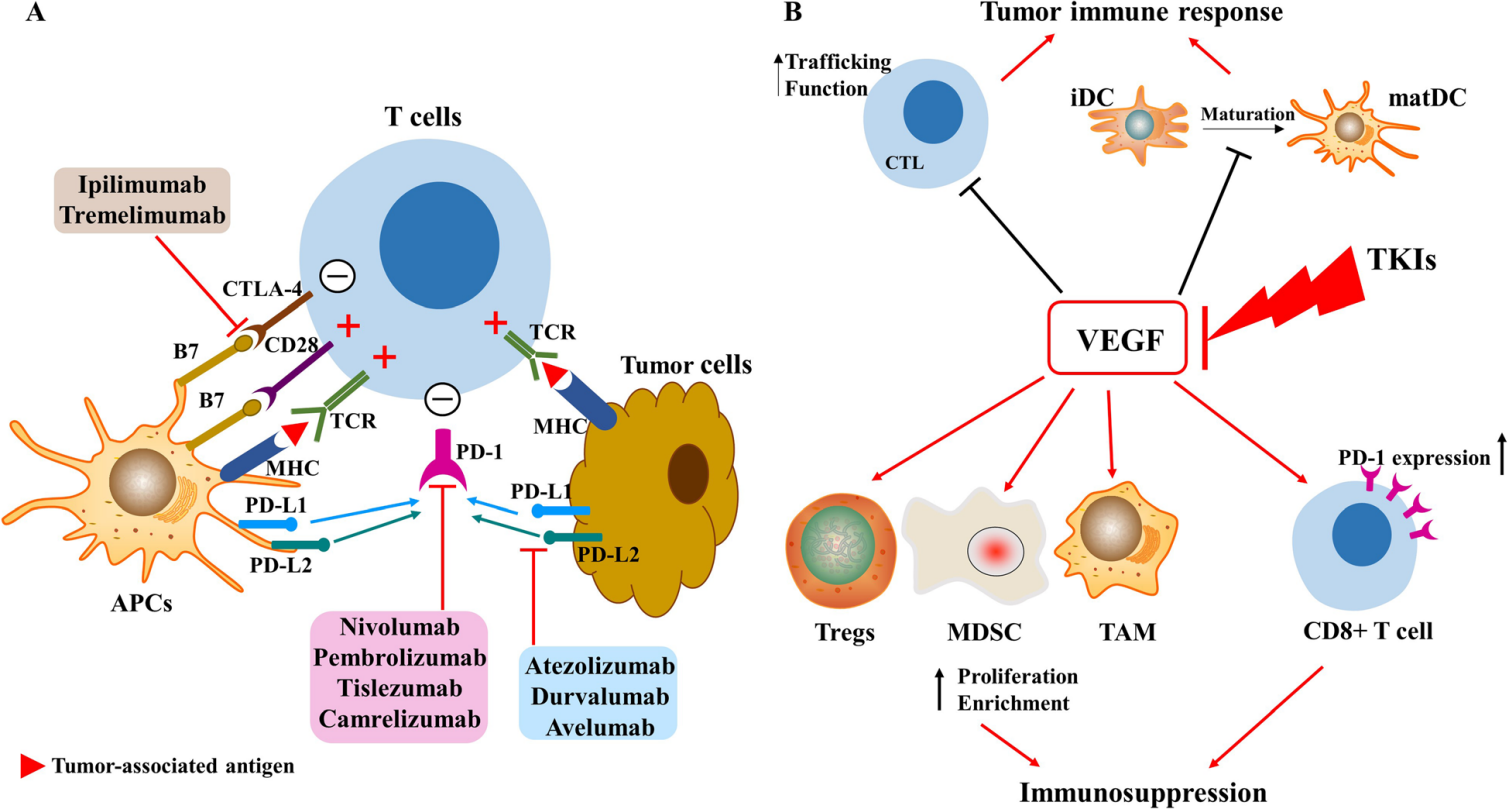
Mechanism of combination therapies. (**A**) Complementary mechanisms of PD-1/PD-L1 and CTLA-4 inhibitors. Presentation of tumor-associated antigen by the major histocompatibility complex (MHC) expressed by APCs results in the release of an activation signal in combination with a co-stimulatory signal via the B7-CD28 pathway, leading to activation of T cells in the lymph node; B7 also binds to CTLA-4 with a higher affinity than that of CD28, in which case T cells cannot be activated. PD-1 on T cells inhibits antigen-specific T cell activation by interacting with its ligands PD-L1 and PD-L2. Immune escape is induced through the PD-1/PD-L1 axis, as well as the B7/CTLA-4 axis. This figure was adapted from Kudo, et al. [44]. (**B**) VEGF modulates the immunosuppressive TME, and TKIs restore this suppressive effect. Red arrows represent promotion effects. APCs, antigen presenting cells; CTL, cytotoxic T lymphocyte; CTLA-4, cytotoxic T-lymphocyte antigen 4; iDC, immature dendritic cell; matDC, mature dendritic cell; MDSCs, myeloid-derived stem cells; PD-1, programmed cell death protein 1; PD-L1, programmed cell death-ligand 1; TAMs, tumor-associated macrophages; TME, tumor microenvironment; Tregs, regulatory T cells. Note: This is an open access article distributed under the Creative Commons Attribution License that permits unrestricted use, distribution, and reproduction in any medium, provided the original work is properly cited (CC BY 4.0)

**Fig. 4:**
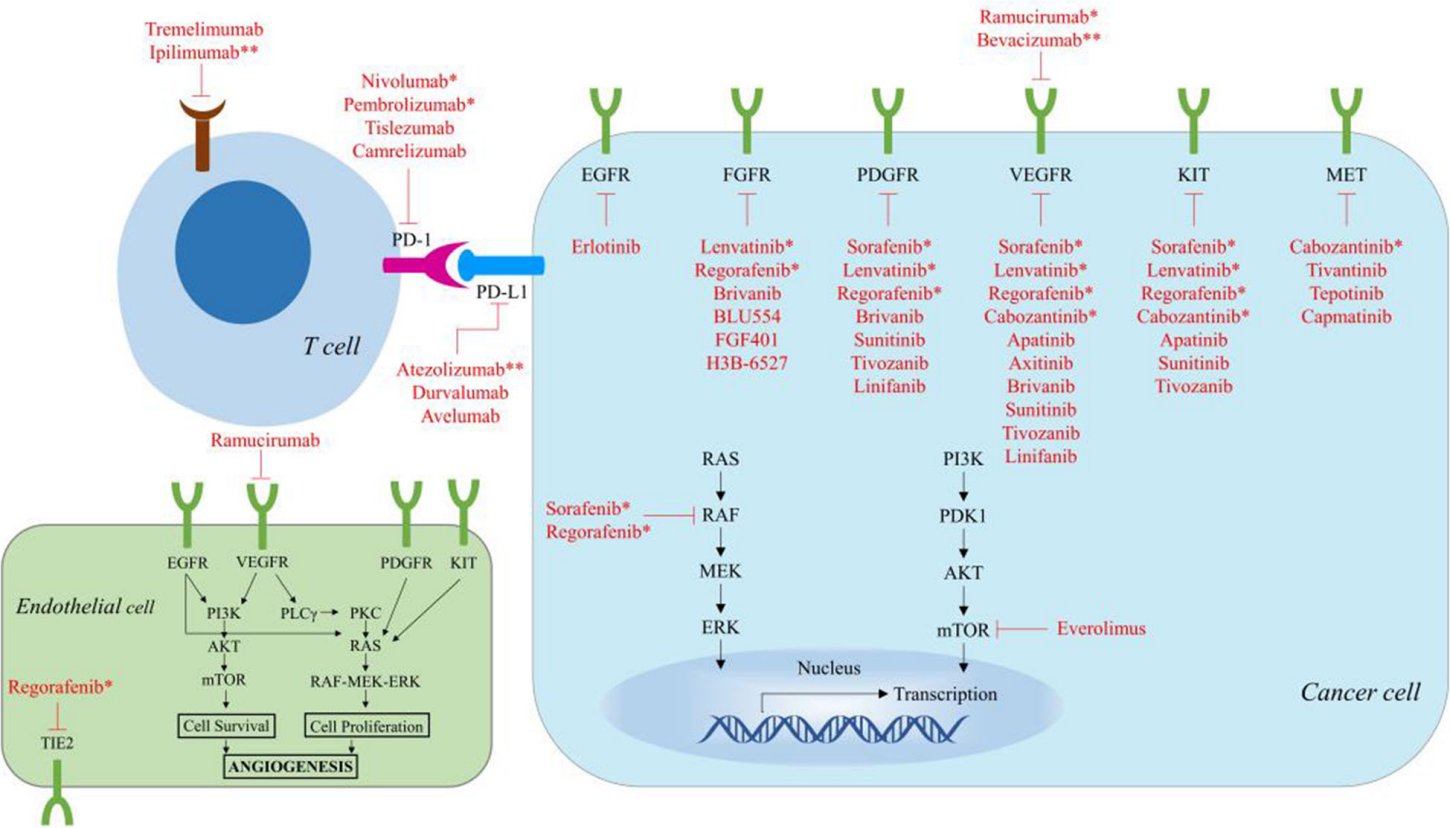
Signaling pathways and molecular targeted therapies for HCC. * represents monotherapies approved by the FDA, ** represents agents as a component of combination therapy approved by the FDA. This figure was modified from Mossenta, et al. [10]. Note: This is an open access article distributed under the Creative Commons Attribution License that permits unrestricted use, distribution, and reproduction in any medium, provided the original work is properly cited (CC BY 4.0)

Nivolumab, a monoclonal antibody, blocks the PD-1 signaling pathway (Figs. 3 and 4) and restores the anti-tumor immune activity. It has been authorized by the U.S. FDA for application in several tumors (e.g., melanoma, non-small cell lung cancer [NSCLC], and kidney cancer) [44]. CheckMate 040 (NCT01658878), a noncomparative phase I/II trial, was designed to investigate the safety and efficacy of nivolumab for advanced HCC patients by escalating and expanding the dose of the medication. Prior sorafenib was allowed. Safety and tolerability represented the primary endpoints for the dose-escalation phase (*n*=48), while ORR was the primary endpoint for the dose-expansion phase (*n*=214). During the dose-escalation phase, the safety profile of nivolumab was evaluated, and the patients’ tolerability of the medication was acceptable: 25% of the patients (12 of 48) had grade 3 or 4 treatment-related adverse events (TRAEs), and the incidence of TRAEs appeared to be dose-independent. In the dose-expansion phase, the ORR was 20% (42 of 214 patients, 95% CI, 15-26), with thirty-nine partial responses and three complete responses. The median duration of response was 9.9 months, and the mPFS was 4.0 months (95% CI, 2.9-5.4). Seventy-nine of 138 patients (57%) had their disease under control, with most disease stabilizations lasting for at least 6 months. Among uninfected untreated patients, the ORR was 23%, and the nine-month overall survival rate was 82%, which supported that nivolumab deserved further investigation as first-line therapy for advanced HCC patients [45]. Based on the ORR and the durable objective responses, on September 22, 2017, the U.S. FDA accelerated the approval of nivolumab to treat HCC patients who were first treated with sorafenib [46].

Another research team studied first-line treatment (nivolumab vs. sorafenib) for advanced HCC patients through a randomized and multicenter phase III study (CheckMate 459; NCT02576509) and reported its data at the 2019 Congress of European Society for Medical Oncology (ESMO). The primary endpoint, OS, was not statistically significant, as the mOS was 16.4 and 14.7 months in the nivolumab group and sorafenib group, respectively (HR=0.85, *p*=0.0752). Interestingly, patients receiving nivolumab had a higher complete response rate (ORR, 15% vs. 7%, respectively). Eighty-one patients (22%) taking nivolumab and 179 patients (46%) taking sorafenib had grade 3/4 TRAEs [47]. Although the above findings may not influence the current standard treatment, they give us insight that immunotherapy may also have a position in the first-line treatment for advanced HCC.

#### Pembrolizumab

Pembrolizumab, a monoclonal antibody blocking PD-1 (Figs. 3 and 4), has demonstrated potent anti-tumor activity and tolerable safety across many cancers [48], and its efficacy and safety were evaluated in advanced HCC patients via a nonrandomized, multicenter phase II trial (KEYNOTE-224; NCT02702414). KEYNOTE-224 revealed that 18 of 104 patients displayed an objective response (17%, 95% CI, 11–26) as per RECIST v1.1, with 1 (1%) achieving a complete response and 17 (16%) achieving a partial response. The median response duration was not reached (range 3.1–14.6+ months), the mTTP and mPFS were both 4.9 months, mOS was 12.9 months (95% CI, 9.7–15.5), and 54% of responding patients had response durations over 12 months [49]. Based on the above data, on November 9, 2018, pembrolizumab received accelerated approval by the U.S. FDA for patients with HCC progressing on sorafenib [50]. At the 2021 ASCO Gastrointestinal Cancer conference, the results of the KEYNOTE-224 cohort 2, which enrolled patients with advanced HCC without prior systemic therapy, were reported: the ORR was 16% (95% CI, 7-29), DCR was 57% (0 CR, 16% PR, 41% SD), mPFS was 4 months (95% CI, 2-6), and mOS was 17 months (95% CI, 8-NA). Based on these results, pembrolizumab monotherapy exhibited durable anti-tumor activity and promising overall survival, supporting further evaluation of pembrolizumab as a first-line treatment for advanced HCC [51].

Following KEYNOTE-224, a double-blind, randomized, phase III study (KEYNOTE-240; NCT02702401) was designed to further assess the safety and efficacy of pembrolizumab treatment. The results of KEYNOTE-240 were updated at the 2021 ASCO GI Conference: the mOS was 13.9 vs. 10.6 months compared to placebo (HR=0.771, 95% CI 0.617–0.964), mPFS was 3.3 vs. 2.8 months compared to placebo (HR=0.703, 95% CI 0.559–0.885), ORR was 18.3% for the pembrolizumab group and 4.4% for the placebo group, DCR was 61.9% vs. 53.3% compared to placebo, and the median TTP was 4.0 vs. 2.8 months [52]. The clinical activity of pembrolizumab was again demonstrated by KEYNOTE-240, confirming the findings from KEYNOTE-224, thus supporting its accelerated approval by the FDA. Two other phase III trials evaluating pembrolizumab as second-line therapy are currently ongoing (KEYNOTE-394 and KEYNOTE-937, Table 2). Pembrolizumab combined with molecular targeted drugs are now being evaluated through several phase I/II trials (Table 3).

### Combination therapy

To achieve a more satisfactory therapeutic response rate than ICI monotherapy, combination therapies based on ICIs are being developed to treat cancer. In 2018, Larkin et al. discovered through the CheckMate067 trial that the use of combined nivolumab and ipilimumab (CTLA-4 inhibitor) was more effective than monotherapy in patients with advanced melanoma [53]. Possible mechanisms for this combination therapy are as follows: 1) blocking the CTLA-4 pathway in lymph nodes increases the activation and proliferation of T cells, leading to CD8+ T cell infiltration; 2) blocking the PD-1/PD-L1 pathway not only maintains the killing capacity of cytotoxic T lymphocytes (CTLs) [44] but also improves the ability of antigen-presenting cells to present tumor-associated antigens; and 3) given the high expression of CTLA-4 and PD-1 on Tregs, the combination of these two inhibitors indirectly reduces the immunosuppressive tumor microenvironment (TME) (Fig. 3A) [54].

Another area of interest is the combination of TKIs with ICIs. It is worth noting that molecular targeted agents (sorafenib, lenvatinib, regorafenib, cabozantinib, and ramucirumab) with beneficial effects on survival in patients with HCC all share the same characteristics, that is, anti-angiogenesis, highlighting the importance of this hallmark in cancer treatment [55]. The restoration of tumor vascular function may help to enhance the ability of other drugs (e.g., ICIs) that can be used in combination with anti-angiogenic molecules to kill tumor cells [56, 57]. TKIs with antiangiogenic activity exert immunomodulatory effects on the TME [58], which include promoting dendritic cell (DC) maturation [59], enhancing T cell trafficking and function [10], and reversing the hypoxia-induced immunosuppressive effect in tissue [60] (Fig. 3B).

#### Nivolumab + Ipilimumab

As discussed above, PD-1 plus CTLA-4 inhibitors promote the anti-tumor immune response through different complementary mechanisms that affect different signaling pathways. Nivolumab in combination with ipilimumab has been shown to be effective in many tumors (e.g., melanoma, NSCLC, renal cell carcinoma, mismatch repair-deficient/microsatellite instability-high metastatic colorectal cancer) [61–64]. In cohort 4 of CheckMate 040 (NCT01658878), 50 patients received 4 doses of nivolumab (1 mg/kg) with ipilimumab (3 mg/kg) every 3 weeks and then 240 mg of nivolumab every 2 weeks (arm A). In this randomized clinical trial, the dosage regimen of arm A showed a good safety profile, a promising objective response rate, and a durable response: the ORR was 32% per RECIST v1.1, with 4 complete responses and 12 partial responses; the median response duration was 17.5 months (4.6–30.5+ months), and 31% of the responses lasted over 24 months [45]. Based on these data, on March 10th, 2020, the dosage regimen of arm A received accelerated approval for advanced HCC patients who progressed on sorafenib treatment [65] (Table 3).

Recently, the 44-month follow-up results from CheckMate 040 were presented at the 2021 ASCO GI Conference. The mOS was 22.2 months in arm A. The 24-month OS rate improved to 46% (95% CI, 32–59), and the 36-month OS rate was 42% (95% CI, 28–55). The ORR remained at 32%, and the median response duration remained at 17.5 months (5–47+ months) in arm A. The DCR was higher in arm A than in arms B and C (54%, 43%, 49%, respectively). Nivolumab + ipilimumab as a second-line treatment continued to show clinical responses and long-term survival benefits [66].

#### Atezolizumab + Bevacizumab

Atezolizumab is an IgG1 monoclonal antibody that specifically binds to PD-L1 and interrupts its interaction with PD-1, thus reversing T cell suppression [67]. Bevacizumab, a humanized anti-VEGF monoclonal antibody, suppresses angiogenesis and tumor development in HCC [68], showing ORRs of 13% [69] and 14% [70] in single-agent phase II trials for advanced HCC. Atezolizumab + bevacizumab showed a tolerable safety profile and efficacy for treating unresectable HCC patients in GO30140 (NCT02715531) [8].

This combination became a promising treatment and is being compared with sorafenib via a phase III trial. In a global, open-label, phase III trial, 501 patients randomly received either atezolizumab + bevacizumab or sorafenib (IMbrave 150; NCT03434379). The efficacy phase showed that the atezolizumab + bevacizumab group had a significantly longer OS and PFS: the estimated 6-month and 12-month survival rates were 84.8% (95% CI, 80.9–88.7) and 67.2% (95% CI, 61.3–73.1), corresponding to 72.2% (95% CI, 65.1–79.4) and 54.6% (95% CI, 45.2–64) for the sorafenib group; the mOS could not be evaluated (NE) in the atezolizumab + bevacizumab group versus 13.2 months (95% CI, 10.4–NE) in the sorafenib group (HR=0.58, *p*<0.001); and the estimated mPFS was 6.8 months and 4.3 months, respectively (HR=0.59, p<0.001). The objective response rates were also statistically significant: the ORR by RECIST v1.1 was 27.3% (95% CI, 22.5–32.5) in the combination group compared to 11.9% (95% CI, 7.4–18.0) in the sorafenib group (*p*<0.001) and 33.2% (95% CI, 28.1 to 38.6) versus 13.3% (95% CI, 8.4 to 19.6) per mRECIST (*P*<0.001). No serious adverse events with significant differences were observed between the two groups. The most common AEs, affecting more than 20% of patients in the atezolizumab + bevacizumab group, were hypertension (29.8%), fatigue (20.4%), and proteinuria (20.1%) [71]. On May 29, 2020, the U.S. FDA approved atezolizumab in combination with bevacizumab as a first-line treatment for advanced HCC patients based on the safety and efficacy revealed in IMbrave 150 [72] (Table 3).

At the 2021 ASCO Gastrointestinal Cancer Symposium, Finn et al. [73] updated the OS analysis for IMbrave 150. After an additional 12 months of follow-up, a sustained clinical efficacy benefit was achieved with atezolizumab + bevacizumab versus sorafenib: the mOS was 19.2 months in the combination group versus 13.4 months with sorafenib (HR=0.66 [95% CI, 0.52–0.85], *P*=0.0009). The updated data showed an ORR of 29.8% (95% CI, 24.8-35) by RECIST v1.1 in the combination group compared to 11.3% (95% CI, 6.9-17.3) in the sorafenib group, and more patients in the combination group achieved a complete response than previously reported (CR=7.7%). This combination regimen has the longest OS ever seen in first-line phase III studies, further confirming that this regimen can serve as a standard of care for patients with advanced HCC who have not received any systemic therapy before.

## Novel therapies in development

### Targeted therapies currently in clinical trials

Currently, five targeted therapies have been approved for the treatment of advanced HCC. Of these five therapies, four are small-molecule kinase inhibitors, and one is a monoclonal antibody against VEGFR2 (Fig. 2). In addition to these approved targeted therapies, a variety of targeted therapies are in clinical development.

#### VEGFR

There are many growth factors involved in tumor angiogenesis, the most important of which is the vascular endothelial growth factor (VEGF) family [74]. In addition to the mentioned inhibitors targeting VEGFR that have been approved by the FDA (sorafenib, lenvatinib, regorafenib, cabozantinib, and ramucirumab), there are several anti-VEGFR agents currently in clinical trials. Tivozanib is a potent VEGFR 1–3 TKI with relatively lesser effects on KIT and PDGFRβ and it has dose-dependent activity against HCC in vivo [75]. According to the existing NCT01835223 data (Table 1) [76], tivozanib showed an ORR (per RECIST v1.1) of 21%, mOS of 9 months (90% CI, 5.4–27.8), mPFS of 24 weeks, and a 1-year OS rate of 40%. Unfortunately, the study did not proceed to stage 2 due to not meeting the prespecified statistical plan. However, tivozanib had better anti-tumor efficacy than sorafenib and lenvatinib as first-line therapy, with ORRs of 21% vs. 6.5% (sorafenib) and 18.8% (lenvatinib), respectively. Since small molecular inhibitors can at least partially reverse immune dysfunction, the combination of tivozanib and ICIs is a promising option. As previously mentioned, a trial of tivozanib in combination with durvalumab is currently ongoing (Table 3).

In addition, at the 2020 ASCO Virtual Scientific Program, two other TKIs designed by Chinese companies reported exciting phase III results. In a first-line treatment setting, donafenib (ZGDH3, NCT02645981, Table 1) had a significantly longer overall survival than sorafenib: the mOS was 12.1 vs. 10.3 months (HR=0.831, *p*=0.0363) (Table 1). Additionally, donafenib exhibited favorable safety and tolerability compared to sorafenib: drug-related AEs of grade ≥ 3 occurred in 37.5% of patients in the donafenib group versus 49.7% in the sorafenib group (*p*=0.0018), and the incidence of treatment interruptions due to drug-related AEs was 30.3% in the donafenib group compared to 42.5% in the sorafenib group (*P*=0.0013) [77].

Apatinib has been investigated as second-line therapy in Chinese advanced HCC patients through a phase III study AHELP (NCT02329860, Table 1), which showed significantly longer OS and PFS compared to placebo and exhibited a tolerable safety profile. The mOS was 8.7 vs. 6.8 months (HR=0.785, *p*=0.0476), the mPFS was 4.5 vs. 1.9 months (HR=0.471, *p*<0.0001), the ORR was 10.7% vs. 1.5% per RECIST v1.1, and no new safety signals were noted [78].

#### c-MET

c-Met is a receptor tyrosine kinase for hepatocyte growth factor (HGF) [79], and abnormalities in c-Met are found in approximately 50% of HCC patients [80]. Aberrant c-MET activity is involved in HCC progression [81] and may lead to treatment resistance toward sorafenib [82]. c-MET inhibitors can be categorized into selective and nonselective. Nonselective c-MET inhibitors (e.g., tivantinib and cabozantinib) may exert anti-tumor effects, mainly owing to the inhibition of non-c-MET targets, and an “off-target” effect is associated with increased toxicity [83]. Conversely, selective c-MET inhibitors (e.g., tepotinib and capmatinib) are being tested in HCC and are thought to have reduced toxicity. Both selective and nonselective agents are being investigated in different trials for potential use in HCC patients as first- or second-line therapy (NCT01988493, NCT02115373; NCT01737827, NCT01964235, Table 1). Decaens et al. reported data of NCT02115373, showing that tepotinib was well tolerated and that the recommended phase 2 dose (500 mg) reached its primary endpoint in the phase 2 study: the 12-week PFS was 63.3% (one-sided binomial exact test: *P* < 0.0001) [84]. Selective c-MET inhibitors are more promising drug candidates. There are also ongoing studies evaluating the safety and tolerability of c-MET inhibitors combined with ICIs (NCT02795429, NCT02082210, Table 3).

#### TGFβ

The TGFβ pathway has dual anti- and pro-tumoral activities in cancer cells: in the early stage, it is beneficial for promoting cell cycle arrest and apoptosis [85], while in the advanced stage, it promotes tumor progression and metastasis by enhancing cell motility, epithelial-to-mesenchymal transition (EMT), invasiveness and stemness [86]. In addition, TGFβ signaling is a major modulator of immune cell proliferation, differentiation, development, and survival [87], which suppresses CD8+ T cells, NK cells, and DCs and increases CD4+CD25+ Tregs by promoting the differentiation of M2-type macrophages [88], leading to immunosuppression in HCC. Microenvironmental remodeling by TGFβ creates a beneficial microenvironment for tumor development and metastasis [86]. These findings provide a rationale for blocking TGFβ signaling in HCC treatment, especially in immunotherapy. Galunisertib (LY2157299) is a TGFβ receptor 1 inhibitor [89] that has been studied as monotherapy and combined with sorafenib in extensive phase I/II trials for HCC [90].

#### Endoglin

Endoglin (also known as CD105), a type 1 intact transmembrane glycoprotein, is a co-receptor for TGF-β ligands and plays an important role in fibrogenesis and angiogenesis [91]. Endoglin is mainly found in immature tumor vascular endothelial cells and contributes to tumor angiogenesis [92, 93]. Endoglin microvascular density has an essential and independent prognostic role in recurrent or metastatic HCC patients [94], and circulating serum endoglin could be detected in HCC patients [93, 95, 96], suggesting that endoglin may be a prognostic marker for HCC patients; however, it still needs further study. TRC105 (carotuximab) is an anti-endoglin antibody, yet it did not show adequate monotherapy efficacy in the phase II trial (NCT01375569) of TRC105 for post-sorafenib HCC patients [97]. The combination of TRC105 and sorafenib (NCT01306058) showed an ORR of 21% per RECIST at all four dose levels, with a mOS of 15.5 months and a mTTP of 3.8 months [98]. At the 2019 ASCO GI, Raghav et al. reported that TRC105 + sorafenib had encouraging signs of activity, with durable partial responses in 1 of 5 patients thus far in phase II (NCT01806064) [99].

#### FGF19/FGFR4

Among the family of tyrosine kinase receptors, fibroblast growth factor receptor 4 (FGFR4) is mostly expressed in the liver [100]. FGF19, one of the three endogenous FGFs, binds with the highest affinity to FGFR4 [101], and the FGF19 expression level is frequently reported to be associated with HCC prognosis [102]. After activation of the FGFR4 signaling pathway, HCC cells undergo proliferation, EMT, anti-apoptosis, angiogenesis, invasion, and drug resistance through activating four downstream pathways: PI3K/AKT/mTOR, RAS/RAF/MAPK, PLCγ/DAG/PKC, and GSK3β/β-catenin [103].. Abnormal expression of FGF19/FGFR4 promotes the development of HCC [102], making it a promising therapeutic target. Despite the relatively low selectivity of pan-FGFR inhibitors against FGFR4 [104], some TKIs specifically targeting FGF19/FGFR4 are being tested in different phases of clinical trials (Table 1) [105], among which the most promising candidate is BLU-554.

In a phase I trial (NCT02508467) to escalate and expand doses, fisogatinib (BLU-554) produced a clinical response in FGF19-positive patients with advanced HCC: the ORR per RECIST v1.1 was 17% (11 of 66 patients) in FGF19-positive patients and 0% in the FGF19-negative group; 53% of TRAEs in this study were grade 1/2, which were tolerable. This trial validates the effectiveness of blocking the FGF19/FGFR4 axis and the biomarker potential of FGF19 to screen HCC patients [106]. Another FGFR4 inhibitor, FGF401, has been investigated as monotherapy or in combination with PDR001 (NCT02325739).

#### CSF-1/CSF-1R

Colony-stimulating factor 1 (CSF-1) is a cytokine mainly produced by tumor cells that recruits macrophages under pathological conditions [107]. When CSF-1/CSF-1R is activated, tumor-associated macrophages (TAMs) secrete growth factors that contribute to tumor growth or metastasis, leading to a higher rate of recurrence [108, 109]. Ao et al. found that PLX3387 (CSF-1R inhibitor, also called pexidartinib) exhibited antitumor activity in both xenograft and allograft HCC models [110], and depletion of TAMs by drugs enhanced the antitumor effects of sorafenib [111]. Several TKIs targeting the CSF-1/CSF-1R axis are now being studied for treating advanced solid tumors, including HCC, such as pexidartinib (NCT02452424), chiauranib (NCT03245190), TPX-0022 (NCT03993873), and BLZ945 (NCT02829723).

### Immune checkpoint therapies in clinical trials

Currently, two immune checkpoint inhibitors have been approved by FDA for the HCC treatment, both of which are PD-1 monoclonal antibodies. In addition to these approved therapies, there are a variety of promising immune checkpoints therapies in clinical trials.

#### PD-1 and PD-L1 inhibitors

In addition to nivolumab and pembrolizumab, there are a number of PD-1 inhibitors and PD-L1 inhibitors in clinical trials, including: (1) PD-1 inhibitors, such as tislezumab versus sorafenib as first-line treatment (RATIONALE 301, NCT03412773), camrelizumab (SHR-1210) as second-line treatment (NCT02989922, 2) PD-L1 inhibitors, namely durvalumab (NCT01693562, NCT03847428), and avelumab (NCT03389126). According to Qin et al. [112], the ORR of camrelizumab was 14.7%, and the 6-month OS was 74.4%. Lee et al. reported the results of a phase II trial of avelumab monotherapy, which demonstrated moderate efficacy and good tolerance with an ORR of 10%, DCR of 73.3%, mTTP of 4.4 months, and mOS of 14.2 months (Table 2) [113]. Except for these two trials, no other trials have reported any results yet.

#### Other immune checkpoint inhibitors

In addition to the well-known PD-1/PD-L1 and CTLA-4, a series of inhibitory immune checkpoint molecules involved in the immune tolerance of HCC have been reported, including lymphocyte activation gene 3 (LAG-3) [114], T cell immunoglobulin mucin-3 (TIM-3)/galectin-9 (GLA-9) [115], T cell immunoglobulin and ITIM domain (TIGHT) [116], and adenosine A2a receptor. Zhou et al. [114] found that a PD-L1 inhibitor in combination with a TIM-3, LAG-3, or CTLA-4 inhibitor results in a revitalization of in vitro tumor-infiltrating lymphocyte (TIL) responses in most patients and further enhances its effect compared to PD-L1 monotherapy. The use of drugs targeting TIM-3/GLA-9 is well summarized in Ref [117]. Next-generation ICIs targeting LAG-3, TIM-3, and TIGHT are expected to provide survival benefits for HCC patients.

#### Agonists of co-stimulatory checkpoint pathways

Immune checkpoints include inhibitory pathways and co-stimulatory pathways. Agonists of co-stimulatory checkpoint pathways such as OX40, GITR, ICOS, CD27, and CD28 are under investigation for treating solid tumors, which have also been summarized in Ref [118]. PF-04518600 is an OX4 agonist antibody. In 2020, the results of a phase I trial (NCT02315066) investigating its safety and tolerability in patients with advanced HCC were published, showing that PF-04518600 was well-tolerated overall and provided meaningful disease control [119]. Glucocorticoid-induced tumor necrosis factor receptor family-related protein (GITR) is believed to promote the function of effector T cells and inhibit the function of Tregs [120]. Beek et al. found that agonistic targeting GITR enhances the function of tumor-infiltrating T cells in HCC, suggesting that it could be a promising target for immunotherapy [121]. Several antibodies targeting GITR including INCAGN01876 (NCT03126110), BMS-986156 (NCT04021043), and TRX518 (NCT02628574, NCT03861403) are being studied for solid tumors involving HCC.

### Combination therapies in clinical trials

Regorafenib enhances the killing effect of T cells by modulating various immunoreactive molecules, helping to present antigens, and triggering T cell differentiation and accumulation [122]. Tumor-associated macrophages associated with angiogenesis in the TME are characterized by TIE2 expression, which is strongly associated with cancer cell intravasation into the circulatory system [123]. This evidence provided a rationale to combine regorafenib and pembrolizumab as first-line treatment for advanced HCC, which is being studied in a multicenter dose-escalation phase IIb trial (NCT03347292, Table 3).

Meanwhile, lenvatinib combined with pembrolizumab has shown promising antitumor activity and manageable toxicity in unresectable HCC through a phase Ib clinical trial (KEYNOTE-524/NCT03006926, Table 3), which supported a Breakthrough Therapy designation granted by the FDA in July 2019 [124]. Efficacy data from KEYNOTE-524 showed an ORR of 46% (95% CI, 36–56.3%) per mRECIST, the mPFS was 9.3 months (95% CI, 5.6–9.7) and mOS was 22 months (95% CI, 20.4–NE) [125]. As a single-arm study, lenvatinib + pembrolizumab from KEYNOTE-524 did not show evidence of meaningful improvement over approved therapies; therefore, a phase III trial is currently assessing the efficacy and safety of this combination compared to lenvatinib + placebo as a first-line option (LEAP-002/NCT03713593, Table 3).

Cabozantinib not only modulates peripheral and intratumor immune landscapes but also induces changes in the phenotype of MC38-CEA tumor cells, thereby increasing their vulnerability to cytotoxic effects mediated by T cells [126]. Immunogenic and immune subset modulations of cabozantinib support its clinical investigation in combination with immunotherapy. Multiple active trials are evaluating the efficacy of cabozantinib in combination with nivolumab/atezolizumab, including the COSMIC-312 phase III trial (NCT03755791, Table 3).

To improve the ORR over single-use nivolumab, a series of different combinations are being tested in clinical trials (Table 3), including sorafenib (NCT03439891), lenvatinib (NCT03418922), BMS-986253 (IL-8 inhibitor, NCT04050462), mogamulizumab (CCR4 inhibitor, NCT02705105), galunisertib (TGFβ inhibitor, NCT02423343) and Pexa-Vec (antitumor vaccine, NCT03071094).

Avelumab (a PD-L1 inhibitor) is being investigated in combination with axitinib/regorafenib via phase 1/2 trials (NCT03289533, NCT03475953, Table 3). VEGF Liver 100 (NCT03289533) is a phase Ib trial to evaluate the safety and efficacy of avelumab + axitinib (VEGFR inhibitor) as a first-line treatment in advanced HCC patients. At the 2019 ASCO GI Cancer, Kudo et al. reported the interim results of VEGF Liver 100: the ORR per mRECIST was 31.8% (95% CI, 13.9–54.9%), and the mPFS was 3.8 months (95% CI, 1.9–7.3) [127].

Camrelizumab was tested in combination with apatinib (VEGFR-2, RET, c-kit inhibitor) for treating advanced HCC patients in NCT02942329 [128]. At the data cutoff (June 15, 2018), 8 of 16 evaluable HCC patients achieved a partial response, with an ORR of 50% (95% CI, 24.7–75.4%). This combination has been evaluated in a phase III trial as a first-line treatment (NCT03764293) versus sorafenib and a phase II trial as a second-line treatment (NCT03463876) (Table 3).

A study investigating tivozanib (selective VEGFR 1–3 TKI) in combination with durvalumab for untreated advanced HCC updated its phase Ib data at the 2021 ASCO GI Conference. NCT03970616 (Table 3) enrolled 7 patients and found that untreated advanced HCC patients tolerated this combination well, with two of them achieving a partial response [129]. The phase II portion of the study is ongoing.

The results of NCT02519348 (Table 3), which evaluated durvalumab (D) combined with tremelimumab (T) in advanced HCC patients, have been reported at the 2020 ASCO Virtual Scientific Program, showing that T300+D (T 300 mg + D 1500 mg 1 dose followed by D Q4 weekly) provided the best benefit-risk profiles. The confirmed ORR by RECIST v1.1 in T300 + D was 24% (95% CI, 14.9–35.3%), mOS was 18.7 months (95% CI, 10.8–NR), and grade 3/4 TRAEs were observed in 26 patients (35.1%) in T300 +D [130]. T300 + D and durvalumab monotherapy are being investigated in the HIMALAYA trial (NCT03298451) as first-line treatment for advanced HCC patients. Besides, a phase III trial evaluating the efficacy and safety of IBI310 (CTLA-4 inhibitor) plus sintilimab (PD-1 inhibitor) versus sorafenib as first-line treatment for advanced HCC is currently recruiting (NCT04720716, Table 3).

### Antibody-drug conjugates (ADCs)

Antibody-drug conjugates (ADCs) consist of a monoclonal antibody against a tumor associated antigen (TAA) on the surface of cancer cells that is conjugated to a cytotoxic drug (known as the payload) through a chemical linker [131]. This directed delivery strategy combines the precision of antibody targeting to antigens with the effective cell-killing activity of the payload, resulting in reduced systemic exposure and thus reduced toxicity [132]. ADCs are one of the rapidly growing areas in cancer drug development, with five ADCs having been approved by the FDA for clinical use (Adcetris, Kadcyla, Besponsa, Mylotarg, and Polivy) and over 100 ADCs being evaluated in clinical trials [133]. The discovery of a series of HCC-specific TAAs provides the rationale for applying ADCs in HCC treatment: glypican-3 (GPC3) [134], AFP, epithelial cell adhesion molecule (EpCAM) [135], tumor endothelial marker 1 (TEM1, or endosialin) [136], MAGE-A, NY-ESO-1 [137], and claudin-6 (CLDN6) [138]. In the past, there were pre-clinical studies of the anti-tumor effects of Anti-CD147 ILs-DOX [139], G7mAb-DOX [140], and MetFab-DOX [141] in HCC, as well as phase I/II trial (NCT01631552) of IMMU-132 [142] in solid tumors including HCC. Recently, Fu et al. designed and synthesized two anti-GPC3 ADCs with duocarmycin SA and pyrrolobenzodiazepine, which were named hYP7-DC and hYP7-PC, and demonstrated that these two ADCs had antitumor activity against both GPC3-positive HCC cell lines and xenograft models [143]. The hYP7-DC was also found to have synergistic effects with gemcitabine, and hYP7-PC induced tumor remission in a mouse model. Kong et al. developed a novel ADC drug, CLDN6-DM1 (DM1 being referred to as mertansine), and the preclinical data of CLDN6-DM1 showed robust antitumor effects against HCC cell lines and a xenograft mouse model, both as monotherapy and combined with sorafenib [144] (Table 4). All these efforts provide a new strategy for the treatment of HCC. Clinical trials of ADCs for HCC treatment in the near future are being anticipated.

**Table 4:** Antibody-drug conjugates and bispecific T cell engagers for HCC treatment

Antibody-drug conjugates				
ADCs	Payload	Target	Stage	Reference
Anti-CD147 ILs-DOX	doxorubicin	CD147	preclinical	[139]
G7mAb-DOX	doxorubicin	CD24	preclinical	[140]
MetFab-DOX	doxorubicin	c-MET	preclinical	[141]
IMMU-132	SN-38	Trop-2	phase I/II	[142]
hYP7-DC	duocarmycin SA	GPC3	preclinical	[143]
hYP7-PC	pyrrolobenzodiazepine			
CLDN6-DM1	mertansine	CLDN6	preclinical	[144]
**Bispecific T cell engagers**				
BiTEs	TAA	T cell receptor	Stage	Reference
1H8/CD3	EpCAM	CD3	preclinical	[145]
solitomab	EpCAM	CD3	phase I	[146]
ERY974	GPC3	CD3	phase I	[147]

Abbreviations: CLDN6, Claudin6; EpCAM, epithelial cell adhesion molecule; GPC3, glypican-3; TAA, tumor associated antigen; Trop-2, trophoblast cell surface antigen-2

### Chimeric Antigen Receptor T Cells (CAR-T)

CAR is a fusion protein consisting of an antigen-binding domain (usually referring to the extracellular portion of the antibody), the signaling domains of the T cell receptor (TCR) chain, and additional co-stimulatory domains (e.g., CD28, OX40, CD137); therefore, CAR recognition is independent of major histocompatibility complex (MHC) restriction. Loss of MHC-associated antigen presentation by tumor cells is a key mechanism of cancer immune escape [148], and one strategy to overcome this escape is to genetically engineer autologous T cells with CAR, expand CAR T cells in vitro and infuse them back into patients to attack cancer cells [149]. In 2017, tisagenlecleucel (KYMRIAH) [150, 151] and axicabtagene ciloleucel (Yescarta) [152, 153] received successive FDA approval as CAR-T therapy for the treatment of acute lymphoblastic leukemia and diffuse large B-cell lymphoma. Breyanzi (lisocabtagene maraleucel) became the third FDA-approved CAR-T therapy in 2021 for certain types of non-Hodgkin lymphoma, including diffuse large B-cell lymphoma [154, 155]. Positive results of CAR-T therapy in hematologic malignancies raised hope for CAR-T therapy against solid tumors [156]. In preclinical studies, engineered GPC3-CAR-T cells could effectively eliminate GPC3-positive HCC cells in HCC xenograft mouse models, demonstrating their clinical potential for treatment [157–159]. There are currently 20 clinical trials in progress testing CAR-T therapy for HCC, 12 of which target glypican-3 (GPC-3) (Table 5). In 2020, Shi et al. [160] reported two sequential phase I studies, NCT02395250 and NCT03146234, which showed that GPC3-CAR-T cells have an initial safety profile and anti-tumor activity in advanced HCC patients. There were two confirmed partial responses of 13 patients, and one patient with sustained stable disease exhibited long-term survival (44.2 months). The 6-month, 1-year, and 3-year OS rates were 50.3%, 42.0%, and 10.5%, respectively, with a mOS of 278 days (95% CI, 48–615 days). There were no grade 3/4 neurotoxicity or CAR-T-related infusion reactions. In addition, CAR-T cells against other antigens are under investigation: anti-c-MET/PD-L1 (NCT03672305 and NCT03638206), anti-DR5 (NCT03941626 and NCT03638206), anti-EGFR (NCT03941626 and NCT03638206), anti-CD147 (NCT03993743), anti-ET1402 (NCT03349255), anti-NKG2D (NCT04550663), anti-mucin1 (NCT02587689), and anti-EpCAM (NCT03013712).

**Table 5:** Ongoing studies investigating CAR-T for HCC

Study Title	Registration ID	Phase	Status	Results/Notes
GPC3-targeted CAR-T Cell for Treating GPC3 Positive Advanced HCC	NCT04121273	1	Recruiting	/
Anti-GPC3 CAR T for Treating Patients with Advanced HCC	NCT02395250	1	Completed	6-month OS:50.3%; 1-year OS: 42.0%; 3-year OS: 10.5%, mOS: 278days (95% CI,48-615 days); 2 PRs, 1 SD
CAR-GPC3 T Cells in Patients with Refractory Hepatocellular Carcinoma	NCT03146234	/	Completed	
Glypican 3-specific Chimeric Antigen Receptor Expressing T Cells for Hepatocellular Carcinoma (GLYCAR)	NCT02905188	1	Recruiting	/
GPC3-CAR-T Cells for the Hepatocellular Carcinoma	NCT04506983	1	Not yet recruiting	/
Anti-GPC3 CAR-T for Treating GPC3-positive Advanced Hepatocellular Carcinoma (HCC)	NCT03084380	1/2	Unknown	/
A Study of GPC3 Redirected Autologous T Cells for Advanced HCC	NCT02715362	1/2	Unknown	/
A Study of GPC3-targeted T Cells by Intratumor Injection for Advanced HCC (GPC3-CART)	NCT03130712	1/2	Unknown	/
GPC3-CAR-T Cells for Immunotherapy of Cancer with GPC3 Expression	NCT03198546	1	Recruiting	/
4th Generation Chimeric Antigen Receptor T Cells Targeting Glypican-3	NCT03980288	1	Recruiting	/
Chimeric Antigen Receptor T Cells Targeting Glypican-3	NCT03884751	1	Recruiting	/
Clinical Study of Redirected Autologous T Cells with a Chimeric Antigen Receptor in Patients with Malignant Tumors	NCT03302403	/	Not recruiting	/
Clinical Study on the Efficacy and Safety of c-Met/PD-L1 CAR-T Cell Injection in the Treatment of HCC	NCT03672305	1	Unknown	/
A Study of CD147-targeted CAR-T by Hepatic Artery Infusions for Very Advanced Hepatocellular Carcinoma	NCT03993743	1	Recruiting	/
Clinical Study of ET1402L1-CAR T Cells in AFP Expressing Hepatocellular Carcinoma	NCT03349255	1	Terminated	Will study new T-cell construct for the same indication
Autologous CAR-T/TCR-T Cell Immunotherapy for Solid Malignancies	NCT03941626	1/2	Recruiting	/
NKG2D CAR-T (KD-025) in the Treatment of Relapsed or Refractory NKG2DL+ Tumors	NCT04550663	1	Not yet recruiting	/
Phase I/II Study of Anti-Mucin1 (MUC1) CAR T Cells for Patients with MUC1+ Advanced Refractory Solid Tumor	NCT02587689	1/2	Unknown	/
A Clinical Research of CAR T Cells Targeting EpCAM Positive Cancer	NCT03013712	1/2	Unknown	/
Autologous CAR-T/TCR-T Cell Immunotherapy for Malignancies	NCT03638206	1/2	Recruiting	/

Clinical trials were searched by keywords “HCC” and “CAR-T” on ClinicalTrials.gov, retrieved on May 1st, 2021. Withdrawn trials were excluded. Abbreviations: mOS, median overall survival; PR, partial response; SD, stable disease

### Bispecific T cell Engager (BiTE)

BiTEs are generated by two different single-chain variable fragments (scFvs) covalently linked by small peptides which are derived from the antigen-binding domains of anti-CD3 and anti-TAA antibodies [161]. BiTEs are believed to possess remarkable efficacy for tumor immunotherapy because of their mini size, high flexibility, and high-affinity binding between effector and target cells [162]. Blinatumomab, an anti-CD19/CD3-BiTE, has been successful in treating hematologic malignancies and was approved by the FDA for treating acute lymphocytic leukemia [163]. Zhang et al. reported that the anti-EpCAM BiTE 1H8/CD3 had effective antitumor activity against HCC cells both in vitro and in vivo [145]. Kebenko et al. [146] reported a phase I study of solitomab (MT110, targeted EpCAM and CD3, NCT00635596) for advanced solid tumors. The study was associated with dose-limiting toxicities, based on which it failed to escalate the dose to potentially therapeutic levels. ERY974 [147], an anti-GPC3/CD3 BiTE, had been evaluated for safety and efficacy in GPC3+ HCC patients in a phase I trial (NCT02748837), with no results yet (Table 4). To improve the efficiency of BiTEs and reduce on-target off-tumor toxicities, the identification of target molecules abundantly expressed by tumor cells compared to nonmalignant cells is necessary. In addition, BiTEs face the complex TME of solid tumors and drug resistance due to the loss of the target antigen expression. These are all key issues to be addressed in the future.

## Suggested systemic treatment strategies

**Fig. 5:**
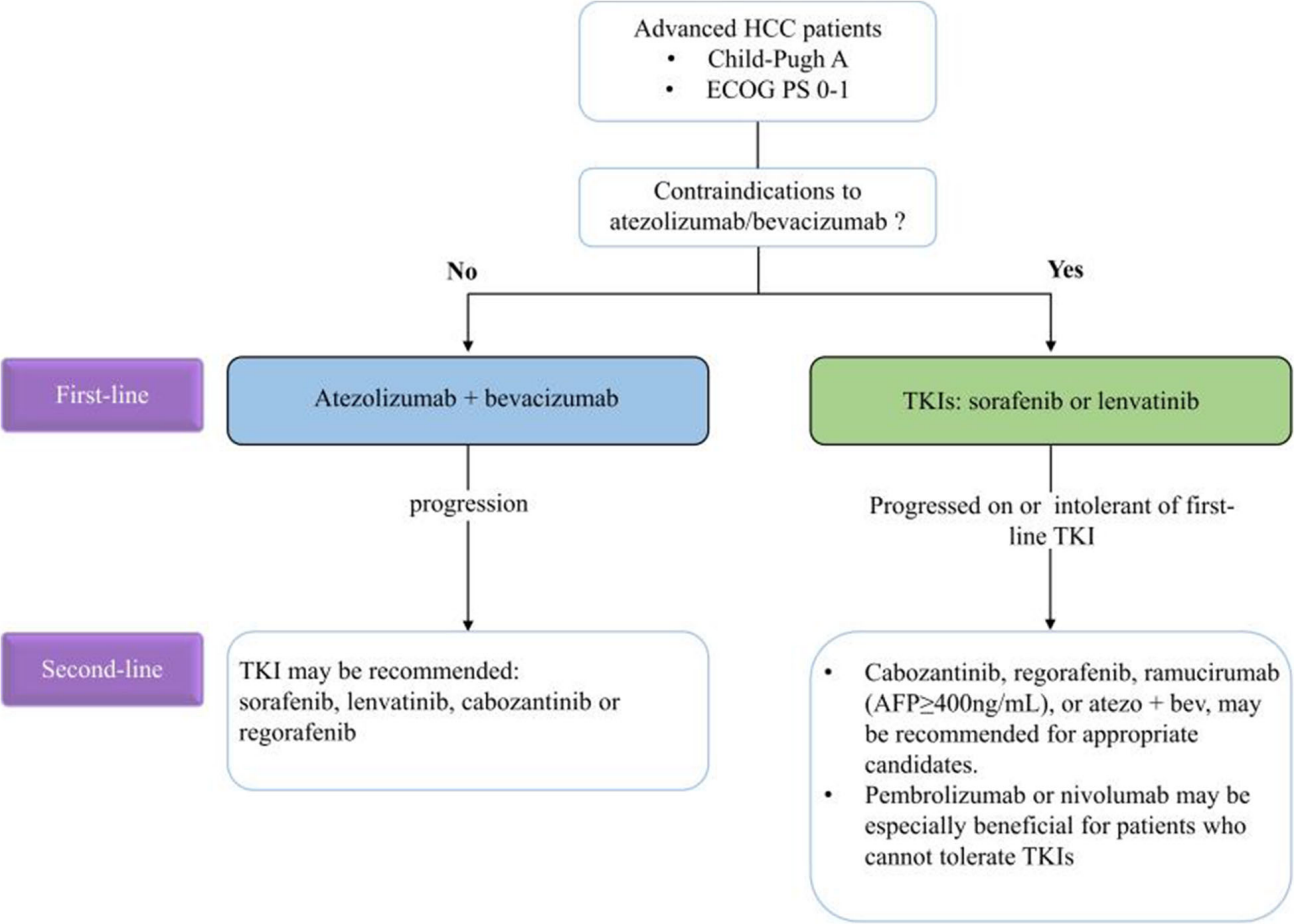
Suggested systemic treatment strategies for advanced HCC. This algorithm is derived from recommendations of “Systemic Therapy for Advanced Hepatocellular Carcinoma: ASCO Guideline” [166]

Recently, approved systemic agents do offer more options for the treatment of advanced HCC patients; however, the lack of head-to-head comparative studies between first- and second-line regimens for systemic treatment has led to confusion when making clinical decisions. Thus, systematic review and network meta-analysis are particularly important at this stage. A meta-analysis of 14 clinical trials conducted by Sonbol et al. showed that atezolizumab + bevacizumab was superior in the first-line setting compared to sorafenib (HR=0.58), lenvatinib (HR=0.63), and nivolumab (HR=0.68). In the second-line setting, all drugs showed a PFS benefit compared with placebo, with only regorafenib and cabozantinib converting to OS benefit. In addition, cabozantinib and regorafenib showed a PFS advantage over ramucirumab and pembrolizumab, but only regorafenib had a statistically significant OS benefit compared to ramucirumab (HR=0.71). For patients with advanced HCC whose AFP levels were at or above 400 ng/mL, there was no significant difference in PFS or OS among regorafenib, cabozantinib, and ramucirumab [164]. Park et al. incorporated 13 first-line studies and 11 second-line studies to obtain similar results. In first-line therapies, atezolizumab + bevacizumab had the greatest OS benefit, while lenvatinib had the greatest ORR benefit. In second-line therapies, cabozantinib had the greatest PFS benefit and ORR benefit compared to placebo [165]. In 2020, ASCO assembled an expert panel to perform a systematic review of published phase III trials and to develop evidence-based clinical practice guidelines for systemic therapy of advanced HCC [166]. According to these guidelines, atezolizumab + bevacizumab is the standard treatment for most patients with advanced HCC in Child-Pugh grade A and Eastern Cooperative Oncology Group Performance Status (ECOG PS) 0–1, the details of which are shown in Fig. 5. For patients with contraindications to immunotherapy, sorafenib or lenvatinib is preferred for first-line treatment.

## Conclusions

Undoubtedly, the systemic treatments of advanced HCC have ushered in prime time (Fig. 4). Angiogenesis and immune evasion are two common features of cancers [167]. As a pioneer in the era of immune combination therapy, the atezolizumab + bevacizumab regimen is the first combination proven to be more effective than sorafenib in the first-line treatment of advanced HCC, which is regarded as a milestone and an encouraging breakthrough in the treatment of advanced HCC. Notably, with atezolizumab + bevacizumab being promoted as a preferred first-line treatment [164–166], the following questions arise: are the existing second-line treatment options still valid? Can the existing first-line TKIs become the second-line treatment after immunotherapy? These points remain to be addressed by future studies. As mentioned earlier, several phase III trials in the first-line setting are underway (e.g., CheckMate-9DW, LEAP2, COSMIC-312, HIMALAYA, Table 3), and the potential first-line treatment options are likely to continue to increase, making clinical decisions more challenging.

Our review not only lists the new drugs approved by FDA for HCC treatment, but also summarizes novel drug candidates under investigation. Selective inhibitors of VEGFR, c-MET, TGFβ, endoglin, and FGFR4 have shown good tolerability and efficacy in phase I/II or phase III studies, making these compounds promising for future treatment. In the field of immunotherapy, in addition to targeting PD-1/PD-L1 and CTLA-4, an increasing number of ICIs, such as LAG-3, TIM-3, and GITR, are gradually demonstrating their efficacy. Meanwhile, as more and more HCC-specific TAAs are identified, other immunotherapeutic approaches, including ADCs, CAR-T, and BiTEs, are being further developed.

The development of new drugs for HCC treatment may not be as impressive as for some other tumors, and few predictive biomarkers have yet been identified (except AFP for ramucirumab and perhaps FGF19 for FGFR4 inhibitors). Based on the results of recent clinical trials, it seems that a single drug may not be sufficient for the treatment of HCC [168], therefore combination therapy represents a major research direction for the systemic treatment of advanced HCC. In addition, it is important to find biomarkers that can predict the treatment response to guide systemic therapy strategy. With the continued development of new therapeutic strategies, it is expected that the treatment outcomes for HCC will be greatly improved in the future.

## Data Availability

The materials supporting the conclusion of this review have been included within the article.

## Abbreviations

ADCs: Antibody-drug conjugates
AEs: Adverse events
ASCO: American society of clinical oncology
BiTE: Bispecific T cell engager
CAR-T: Chimeric antigen receptor T Cells
CI: Confidence interval
CTL: Cytotoxic T lymphocyte
CTLA-4: Cytotoxic T lymphocyte associated antigen 4
DCR: Disease control rate
FGF: Fibroblast growth factor
FGFR: Fibroblast growth factor receptor
HCC: Hepatocellular carcinoma
HR: Hazard ratio
ICIs: Immune checkpoint inhibitors
MDSCs: Myeloid-derived suppressor cells
ORR: Objective response rate
OS: Overall survival
PDGFR: Platelet-derived growth factor receptor
PFS: Progression-free survival
RECIST: Response evaluation criteria in solid tumors
TAMs: Tumor-associated macrophages
TKIs: Tyrosine kinase inhibitors
TME: Tumor microenvironment
TRAEs: Treatment-related adverse events
Tregs: T-regulatory cells
TTP: Time to progression
VEGFR: Vascular endothelial growth factor receptor
